# Respiratory function monitoring during neonatal resuscitation: a crossover study of high-fidelity simulated bronchopulmonary dysplasia

**DOI:** 10.3389/fped.2026.1940755

**Published:** 2026-10-07

**Authors:** Allan Jenkinson, Kerstin Jost, Janneke Dekker, Emma Williams, Almudena Alonso-Ojembarrena, Anna Lavizzari, Benjamin Stoecklin, Olivier Danhaive, Theodore Dassios

**Affiliations:** 1 Department of Women and Children’s Health, King’s College London, London, United Kingdom; 2 Department of Women’s and Children’s Health, Karolinska Institute, Stockholm, Sweden; 3 Division of Neonatology, Department of Paediatrics, Willem-Alexander Children’s Hospital, Leiden Universitair Medisch Centrum, Leiden, Netherlands; 4 Neonatal Intensive Care Unit, Chelsea and Westminster Hospital, London, United Kingdom; 5 Neonatal Intensive Care Unit, Puerta del Mar Universitario Hospital, Cádiz, Spain; 6 Fondazione IRCCS Ca’ Granda Ospedale Maggiore Policlinico, Milan, Italy; 7 Department of Neonatology, University Children’s Hospital Basel (UKBB), Basel, Switzerland; 8 Division of Neonatology, Saint-Luc University Hospital, UCLouvain, Brussels, Belgium; 9 Neonatal Intensive Care Unit, University of Patras, Patras, Greece

**Keywords:** bronchopulmonary dysplasia, high-fidelity simulation, manikin, neonatal resuscitation, tidal volume

## Abstract

**Purpose:**

We aimed to test the hypothesis that respiratory function monitoring (RFM) during neonatal resuscitation can reduce mask leak and the delivery of inadvertently high tidal volumes.

**Methods:**

We used a high fidelity neonatal manikin mimicking the lung mechanics of bronchopulmonary dysplasia. Neonatal clinicians provided positive pressure ventilation with a bag-valve and mask while blinded and while unblinded to the RFM, displaying the leak and the tidal volume.

**Results:**

The median (IQR) inspiratory tidal volume when blinded to the RFM [8.3 (7.1–10.6) ml/kg] was significantly higher compared to when unblinded to the RFM [6.7 (5.8–8.3) ml/kg, *p* = 0.002]. The expiratory tidal volume when blinded to the RFM [6.5 (5.8–7.8) ml/kg] was significantly higher compared to when unblinded to the RFM [5.7 (5.1–6.9) ml/kg, *p* = 0.021]. The percentage of leak was not different when blinded to the RFM compared to when unblinded. The peak inflation pressure was not different when blinded to the RFM compared to when unblinded.

**Conclusion:**

During simulated resuscitation of bronchopulmonary dysplasia, participants were more likely to achieve a tidal volume within the intended range and a higher respiratory rate when they had real-time RFM feedback compared to when such feedback was not available.

## Introduction

Assisting the transition from fetal to postnatal life at birth is a common intervention practiced widely by health practitioners in newborn care (1, 2). More rarely, some degree of resuscitation might be required outside the delivery ward, for hospitalised premature infants with evolving or established bronchopulmonary dysplasia (BPD). Infants with BPD have increased airway resistance necessitating ventilation and resuscitation using prolonged inflation times and higher inflation pressures (3).

The efficacy of ventilation during resuscitation is clinically assessed with the subjective marker of the “chest rise”, indicating that air is entering the lungs (2). Although visible chest movement is recognised as an indicator of lung inflation, and absent chest movement calls for corrective measures for ineffective ventilation, visual assessment cannot quantify the actual delivered tidal volumes which might be above or below the intended target range. Furthermore, resuscitators are mostly unable to accurately assess the chest wall movement using visual inspection only (4). One way towards providing a more personalised form of “volume-targeted resuscitation” is the use of a respiratory function monitor (RFM) and the continuous real-time display of the tidal volume and mask leak (5). We have previously used an RFM and high-fidelity simulation with normal lung mechanics to report an improvement in the mask leak (6).

In this study, we aimed to use high-fidelity simulation with real-time respiratory function monitoring to investigate if using the RFM decreased the leak and improved tidal volumes during bag-valve-mask ventilation during resuscitation in a scenario of simulated BPD.

## Methods

Delegates and faculty participating in the Research Workshop on Neonatal Pulmonology of the European Society for Paediatric Research (ESPR) took part in the study. The workshop was organised by the Pulmonology and Early Career sections of the ESPR and took place in Corfu, Greece, in May 2026. Participants provided informed consent to participate before starting the workshop.

### The respiratory function monitor and simulation manikin

The Monivent Neo100 is a compact wireless RFM for real-time assessment of the efficiency of mask ventilation during neonatal resuscitation. The RFM operates with an integrated differential pressure pneumotachometer, connected to a face mask and a resuscitator bag with a capacity of 500 mL and a safety pressure-relief valve at 40 cmH_2_O (Figure 1). The pneumotachometer introduces an added deadspace of <1.5 mL, corresponding to the sensor mask and the mask adapter. The measurement accuracy for the expiratory tidal volume is ± 1 mL or 10% of the reading and the RFM autocalibrates at start-up. The RFM provided continuous real-time display of the tidal volume (mL/kg), peak inspiratory pressure (cmH_2_O), peak end-expiratory pressure (cmH_2_0), leak (%) and inflation rate (breaths per minute). The Lusi Neonatal Lung Simulator (NeoSim, Switzerland), with the windows-based editing software LuSiLIFE was used with lung pathology settings corresponding to the respiratory mechanics of BPD with a resistance of the respiratory system of 400 cm H_2_O/L/s and compliance of the respiratory system of 1.0 mL/cm H_2_O (7).

**Figure 1:**
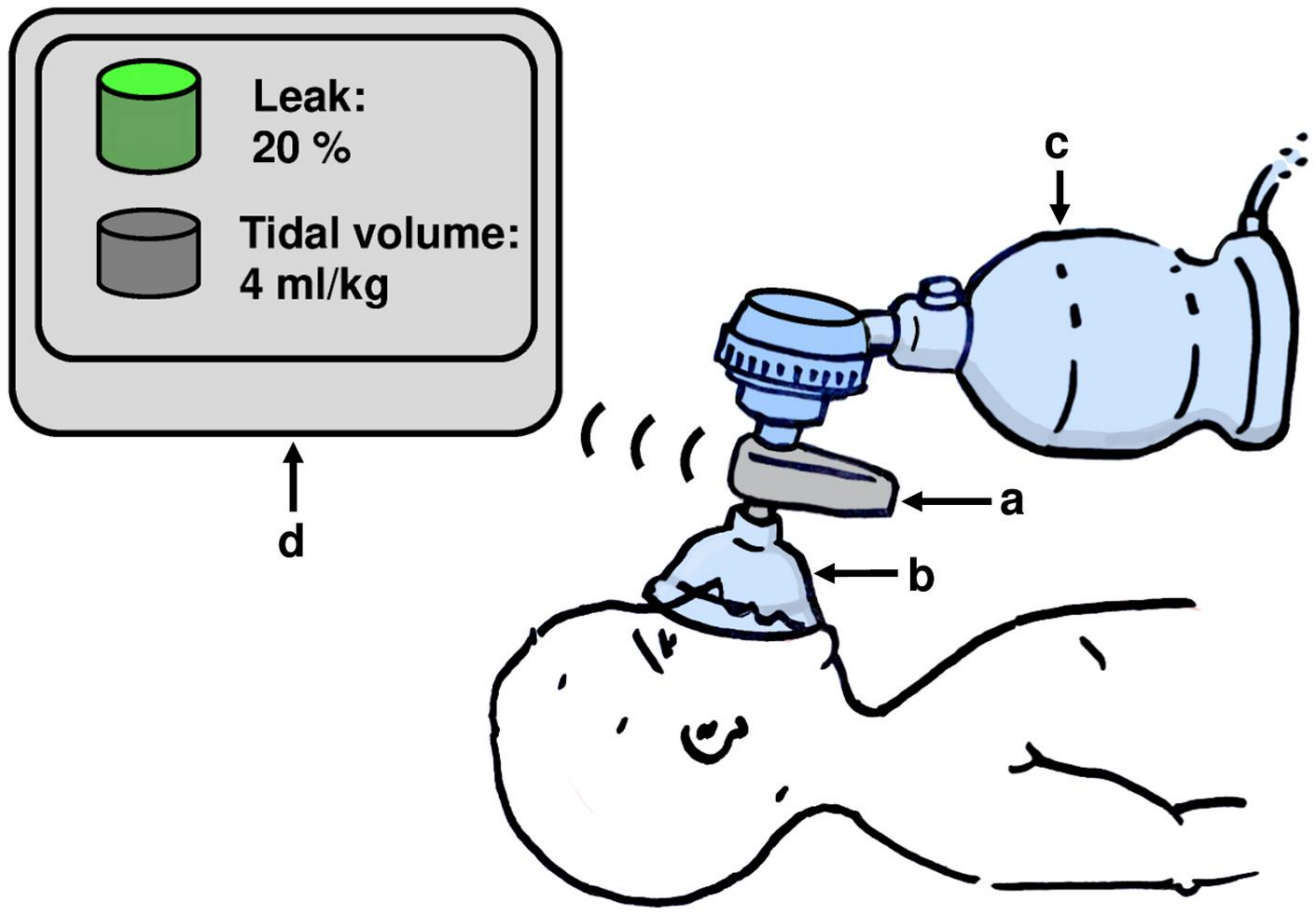
Position of the wireless respiratory function monitor **(a)**, between the face mask **(b)** and resuscitator bag **(c)**, and the screen **(d)** presenting the % leak and delivered tidal volume to the simulation manikin.

### Procedure

After a training period with a scripted explanation, instructor-led demonstration and hands-on familiarisation, participants were asked to provide positive-pressure ventilation via a bag-valve and mask system. In the absence of specific guidelines for the resuscitation of infants with BPD, participants were asked to provide resuscitation as per the European Resuscitation Council guidelines for newborn resuscitation (2), delivering five inflations, each of 2–3 s duration followed by 30s of ventilation breaths, aiming to achieve a tidal volume of 5–8 mL/kg (8), a rate of 60 breaths per minute and a mask leak of 0%. The participants were asked to perform this sequence twice: first with the participant blinded to the RFM display followed by a second sequence when the participants were unblinded to the RFM. Data from the RFM on the tidal volumes, leak, peak inflation pressure and breathing rates were recorded in real time on an Excel spreadsheet. The mean value of all the results from the blinded and the unblinded sequences were calculated. The tidal volumes were adjusted for a body weight of 2.5 kg according to the simulation manikin manufacturer specifications.

### Statistical analysis

Continuous data were tested for normality with the Kolmogorov–Smirnov test. Non-normally distributed data were presented as median and interquartile range (IQR). The tidal volumes, leak, peak inflation pressure and breathing rates during the ventilation breaths while blinded to the RFM and when unblinded to the RFM were compared using the Wilcoxon signed rank test. To establish the relation of previous clinical experience with the benefit of using the RFM, the improvement of the percentage of leak was compared in the three categories of clinical experience (less than six months, six months to two years, and more than two years) using Kruskal Wallis testing. The significance level was *p* < 0.05. Statistical analysis was performed using IBM SPSS v.29 Software (IBM, Chicago, IL).

### Sample size justification

The median leak in 23 infants of a median gestation of 38 weeks was 30% (IQR: 11%–44%) (9). From this IQR the standard deviation of the leak was calculated at 12% (10). To detect a decrease in the leak by 50% with a power of 80% and significance of 0.05, a total of 20 paired interventions would be required (9).

## Results

Twenty-five neonatal practitioners participated in the study. Their level of experience ranged from less than six months (*N* = 4), between six months and two years (*N* = 4) and more than two years (*N* = 17).

The median (IQR) *inspiratory* tidal volume when blinded to the RFM [8.3 (7.1–10.6) mL/kg] was significantly higher compared to when unblinded to the RFM [6.7 (5.8–8.3) mL/kg, *p* = 0.002, Table 1]. The *expiratory* tidal volume when blinded to the RFM [6.5 (5.8–7.8) mL/kg] was significantly higher compared to when unblinded to the RFM [5.7 (5.1–6.9) mL/kg, *p* = 0.021, Figure 2, Table 1]. When blinded to the RFM the expiratory tidal volume was outside the recommended range of 5–8 mL/kg in seven participants (five with a value of >8 mL/kg) compared to three participants when unblinded to the RFM (two with a value of >8 mL/kg). The *respiratory rate* when blinded to the RFM [40 (31–53) bpm] was significantly lower compared to when unblinded to the RFM [45 (34–57) bpm, *p* = 0.003, Table 1]. The *peak inflation pressure* was not different when blinded to the RFM [29 (24–35) cmH_2_O] compared to when unblinded [27 (23–34) cmH_2_O, *p* = 0.34, Table 1]. The percentage of leak was not different when blinded to the RFM [10 (7–25) %] compared to when unblinded [8 (6–24) %, *p* = 0.93, Table 1].

**Table 1:** Measured parameters when the participants were blinded and unblinded to the RFM. Values are presented as median (interquartile range).

	Blinded to RFM	Unblinded to RFM	*p* value
Inspiratory tidal volume (mL/kg)	8.3 (7.1–10.6)	6.7 (5.8–8.3	0.002
Expiratory tidal volume (mL/kg)	6.5 (5.8–7.8)	5.7 (5.1–6.9)	0.021
Respiratory rate (bpm)	40 (31–53)	45 (34–57)	0.003
Peak inflation pressure (cmH_2_O)	29 (24–35)	27 (23–34)	0.34
Leak (%)	10 (7–25)	8 (6–24)	0.93

**Figure 2:**
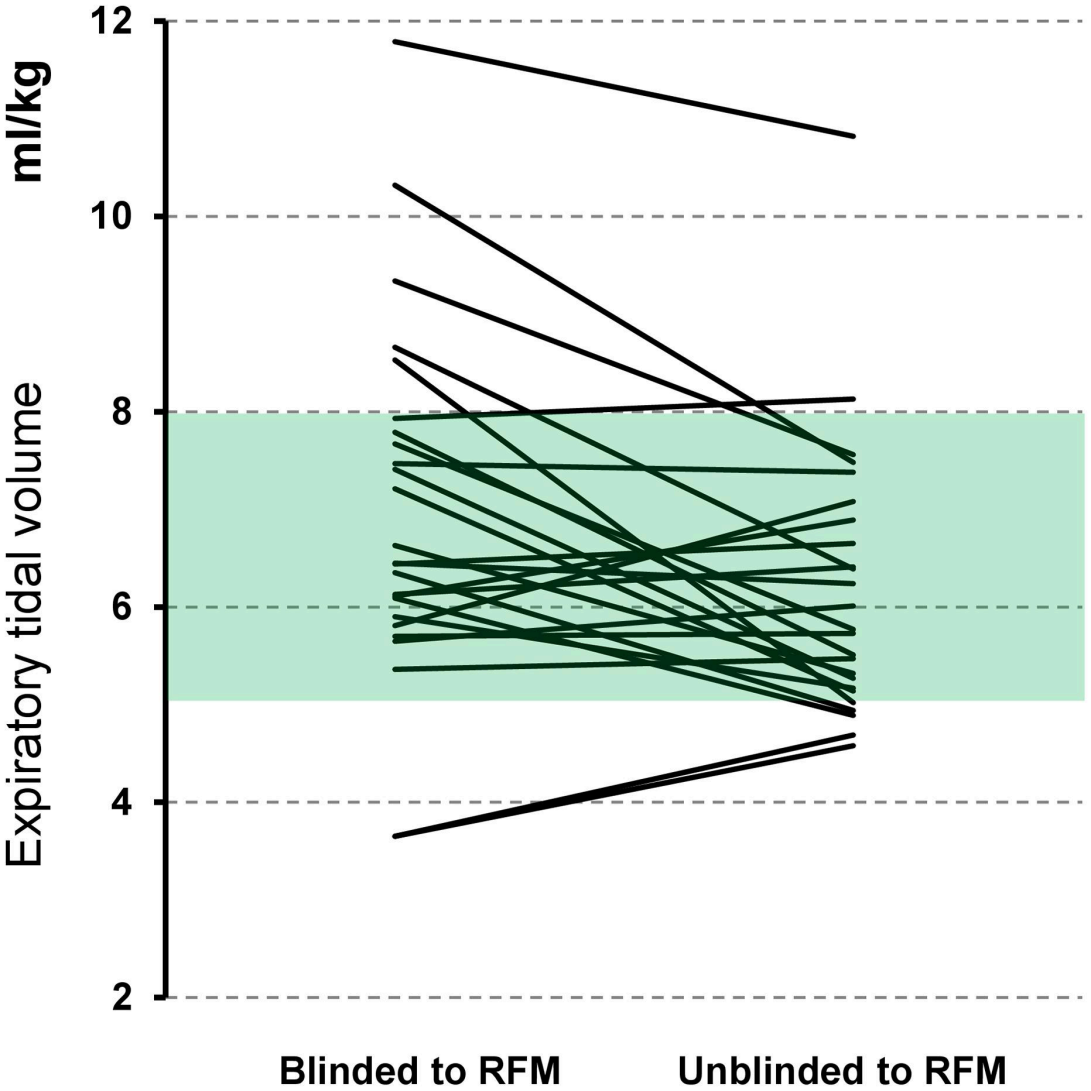
Individual values and direction of change of the expiratory tidal volume when blinded to the RFM and when unblinded to the RFM. The highlighted area represents the range of the targeted values between 5 and 8 mL/kg.

The change in the percentage of leak when blinded to the RFM minus when unblinded to the RFM was 2 (−6 to 12) % and was not different between the groups of participants according to clinical experience. The median (range) values of the change in the percentage of leak were 3 (−4 to 8), 1 (−6 to 3) and 2 (−5 to 12) % for clinical experience of less than six months, six months to two years, and more than two years respectively (*p* = 0.137).

## Discussion

We demonstrated that during simulated resuscitation of infants with BPD, participants were more likely to achieve a tidal volume within the intended range and a higher respiratory rate when they had real-time RFM feedback compared to when such feedback was not available. A previous study of forty-five infants with a median gestation of 38 weeks resuscitated in the delivery room with a T-piece device, providing a fixed peak inflation pressure of 30 cmH_2_O reported a mean expiratory tidal volume of 4.6 mL/kg (11). Similarly, in a population of 129 term newborns resuscitated using a T-piece device and a peak pressure of 30 cmH_2_O the median tidal volume after birth was 4.5 mL/kg (12). These studies differ compared to ours as they used a preset peak pressure and refer to the transitional phase after birth which involves a gradual clearance of lung fluid and establishment of functional residual capacity (13). In our study and despite not using a T-piece device, the median inflation pressures were below 30 cmH_2_O both when blinded and unblended to the RFM, although our study was undertaken in simulated BPD where such recommended pressure cut-offs have not been proposed.

In our study it was interesting to note that more appropriate (lower) tidal volumes were achieved when unblinded to the RFM, without a significant difference in the leak or the inflation pressures. It is possible, that this was due to better positioning of the head and optimising the alignment of the airways responding to real –time RFM feedback. We did not report a different percentage of leak when using the RFM compared to not, and our median leak was only 8%–10%. This contrasts previous resuscitation studies with full-term infants which have reported a median leak of 30% (9). In these studies, however, different equipment and preset pressures were used and the studies were performed by paediatric trainees, while in our study the participants had substantially more clinical experience in resuscitation. The small leak in our study might be explained by the use of this specific simulation manikin, as we have previously used the same manikin and reported median leaks of 13%–16% in a similar population of healthcare providers (6). We should explicitly acknowledge, however, that our primary hypothesis and sample size calculation also included the leak as a targeted parameter and our results, thus *only partially* support the hypothesis.

In our study we selected to aim for a higher range of tidal volumes, going up to 8 mL/kg in an effort to mimic the inherent pathophysiological characteristics of BPD. In the absence of specific recommendations for the use of pressure and inflation times in BPD, we used, however, standard resuscitation pressure and inflation times which are not specifically recommended for infants with BPD (2). It is possible that infants with BPD might require longer inflations and the use of variable pressures to achieve the targeted tidal volumes in the background of inhomogeneous lung disease. Unfortunately, as our lung simulator treated the lungs as a single compartment with a sole value for compliance and resistance, this heterogeneity could not have been captured by our study. Possibly, future studies with live infants might address this point, but they will also have to overcome other major limitations such as for the example the unforeseen and rare nature of an acute deterioration which will necessitate resuscitation in BPD.

We highlighted that the use of RFM can fine-tune resuscitation with more appropriate tidal volume delivery while not exceeding the safe inflation pressure cut-offs. Whether this level of fine-tuning can translate to improved clinical outcomes is a conclusion which falls outside the scopes of this study. Our study was the first to combine the use of RFM and high-fidelity simulation of BPD during neonatal resuscitation. Although our participants were fairly experienced, there was a clear decrease in the used tidal volumes when using the RFM, highlighting a potential lung-protective effect. Our results would suggest that similar simulation-driven data could be used for training to inform resuscitation scenarios and enhance protective ventilation in the context of BPD.

In our study most participants had significant experience in neonatal resuscitation with 17 of 25 reporting more than two years of clinical experience. This might limit the generalisability of our findings as more junior staff and trainees with less clinical experience might benefit more from RFM feedback, especially since they would likely start from higher values of leak when blinded to the RFM display compared to more senior clinicians. Unfortunately, we could not use a T-piece device in our study, however, the use of a bag-valve and mask system allowed us to confirm that a lower pressure can still achieve the required tidal volumes in a scenario of increased airway resistance. Although a fixed pressure device has been reported to be superior to inflation bags in reducing complications such as BPD, this conclusion was based on evidence of low-moderate certainty and refers mostly to preterm infants at birth, while numerous infants internationally are still resuscitated using bag-valve and mask systems (14). Finally, in our study we used a non-randomised, standard order of blinded followed by unblinded inflations. We should acknowledge that this constant order might have introduced an element of order bias favouring the unblinded approach, since the participants possibly practiced and familiarised with the equipment during the blinded phase. We hope that the initial familiarisation phase, however, should have minimised this effect, while we should also state that the unblinded approach would be generally considered “standard practice” with which the participants would be already broadly familiar from previous experience.

In conclusion, when using respiratory function monitoring during resuscitation of simulated bronchopulmonary dysplasia participants were more likely to achieve a tidal volume within the intended range and a higher respiratory rate compared to when such feedback was not available.

## Data Availability

The raw data supporting the conclusions of this article will be made available by the authors, without undue reservation.
